# P06-10 To what extent is active mobility practiced by adults in Italy?

**DOI:** 10.1093/eurpub/ckac095.095

**Published:** 2022-08-29

**Authors:** Valentina Minardi, Valentina Possenti, Benedetta Contoli, Susanna Lana, Stefano Campostrini, Giuliano Carrozzi, Angelo D'Argenzio, Pirous Fateh-Moghadam, Mauro Ramigni, Massimo Oddone Trinito, Maria Masocco

**Affiliations:** 1 National Centre for Disease Prevention and Health Promotion, Istituto Superiore di Sanità, Rome, Italy; 2 Department of Economics, University Ca' Foscari, Venice, Venice, Italy; 3 Department of Public Health, LHU Modena, Modena, Italy; 4 Health Division, Campania Region, Naples, Italy; 5 Department of Prevention, LHU Trento, Trento, Italy; 6 Department of Prevention, LHU 2 Marca Trevigiana, Treviso, Treviso, Italy; 7 Department of Prevention, LHU Rome 2, Rome, Italy

**Keywords:** Active mobility, behavioural surveillance, adult population

## Abstract

**Background:**

Walking or cycling regularly instead of using motorised vehicles returns benefits not only to our health but also to the environment: in Europe, during 2020 a spotlight has also been put on the importance of accessibility to zero-emission transport, for promoting an inclusive framework that involves everyone. Policies in favour of a diffused active mobility in the general population encourage also to take steps effectively in order to achieve the longer-term goal of a European continent that is carbon-neutral.

**Methods:**

In the Italian Behavioural Risk Factor Surveillance System PASSI, active mobility identifies both adults (aged 18-69) who cycle or walk to go to work or to school or for their usual commuting and those who, thanks to this habit, reach out recommended levels of physical activity to gain health benefits. Basing on their own active mobility levels, people are classified in: physically active (they reach out at least 150 minutes per week by walking or cycling for usual commuting, in bouts of at least 10 minutes); partially active (they use bicycle and/or walk usually, but not till 150 minutes weekly); non-active (they do not practise any active mobility or they do for little time duration).

**Results:**

PASSI data 2016-2019 show that 44% among adults residing in Italy has practised active mobility by cycling (11%) and/or walking (41%) for usual commuting. They do in average for 4-5 days per week: people who cycle and those who walk sum up an average of, respectively, 144 and 181 minutes weekly. In the North, active mobility is experienced more than in the other parts of the Country. Active mobility definitively contributes to reach out recommended levels of physical activity that ensure health benefits and, in Italy, 21% of the resident adult population results to be physically active just thanks to this healthy lifestyle.

**Conclusions:**

Walking or cycling for urban commuting, at least for 150 minutes per week in bouts of 10 minutes each, can help to meet the recommendations for physical activity by the WHO, without counting movement in spare time or at work.

